# An efficient and safe process for the preparation of ticagrelor, a platelet aggregation inhibitor via resin-NO_2_ catalyzed formation of triazole ring

**DOI:** 10.1186/s40064-015-1299-6

**Published:** 2015-09-15

**Authors:** Gorakshanath B. Shinde, Pravin K. Mahale, Santhosh A. Padaki, Navnath C. Niphade, Raghunath B. Toche, Vijayavitthal T. Mathad

**Affiliations:** Department of Process Research and Development, Megafine Pharma (P) Ltd., 201, Lakhmapur, Dindori, Nashik, 422 202 Maharashtra India; Department of Chemistry, Organic Chemistry Research Center, KTHM College, Gangapur Road, Nashik, 422002 India

**Keywords:** Ticagrelor, Diazotization, Resin-NO_2_, Triazole, Nitration, Arterial thrombosis

## Abstract

An efficient, safe and improved process for the preparation of ticagrelor **1**, a platelet aggregation inhibitor is described. Synthesis comprises the condensation of pyrimidine amine derivative **14** with cyclopentyl derivative **13** in ethylene glycol followed by construction of triazole compound **16** by diazotization of the obtained intermediate **15** with a green and safer reagent “Resin-NO_2_” in water and acetonitrile mixture. Condensation of **16** with cyclopropylamine derivative **10** followed by deprotection of compound **12** with hydrochloric acid in dichloromethane (DCM) furnished ticagrelor **1** with an overall yield of 65 % and purity of 99.78 % by HPLC. Each reaction stage was optimized independently to establish the scalable and plant friendly process. An efficient and a safe process for key intermediate **14** which involve nitration reaction has also been developed. Safety parameters were established by understanding the thermal events of the reaction by DSC analysis.

**Graphical abstract:**
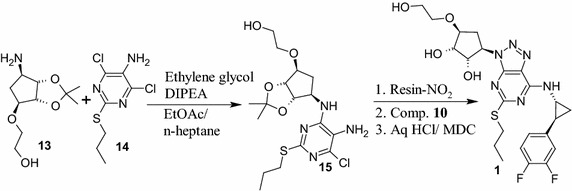
Synthesis of ticagrelor via resin-NO_2_ catalysed formation of triazole ring

Synthesis of ticagrelor via resin-NO_2_ catalysed formation of triazole ring

## Background

Establishing the safe chemical processes for manufacturing of active pharmaceutical ingredients and their intermediates in pharmaceutical/chemical industry is increasingly becoming important and challenging. Identification of critical process parameters by assessing the hazardous factors associated with material, reaction and operation that bring safety to the product and personnel involved in the production is very critical and important to grow and sustain in the business. Accidents can be avoided just by understanding the heat of reaction, kinetics, decomposition pattern of product mixture, addition pattern of reagents and reactants, mischarging of reactants and catalysts, agitation problems, proper temperature control, and etc. In this communication, we presented process development aspects of ticagrelor **1**, a P2Y12 receptor antagonist (Jacobson and Boeynaems 2010) and a well-known dual ADP receptor inhibitor used in the treatment of arterial thrombosis (Springthorpe et al. 2007). This drug was invented by Astra Zeneca and approved by United state of food and drug administration (USFDA) during March 2012 for the prevention of thrombotic events such as stroke or heart attack in patients with acute coronary syndrome or myocardial infarction with ST elevation (Zhang et al. 2012). Through its direct and reversible mode of action, ticagrelor exhibits rapid onset and offset effects and thus more suitable for patients who are going to take surgery (Haberfeld 2010). In addition ticagrelor does not require metabolic activation (Van Giezen and Humphries 2005), indicating less variability related to genetic polymorphism (Huber et al. 2011).

**Scheme 1:**
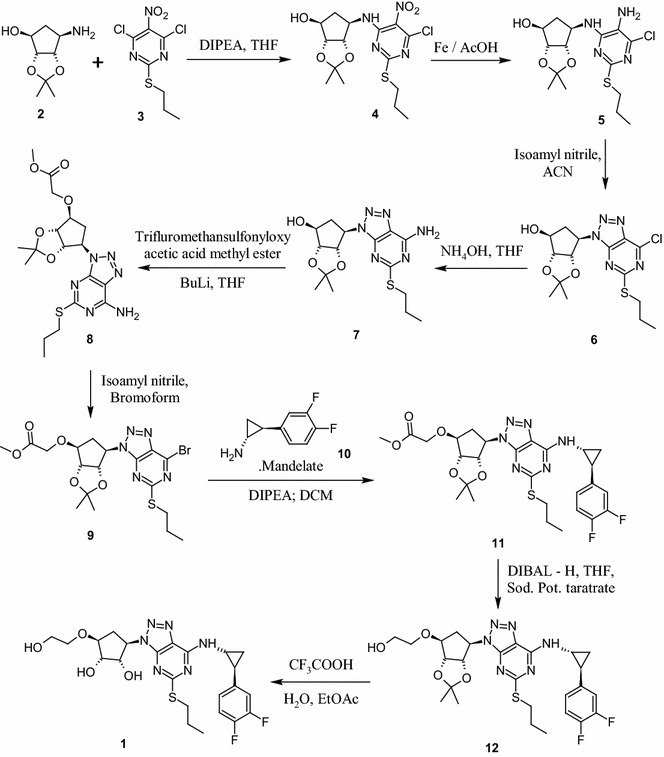
Reported process for ticagrelor as per basic patent route

**Fig. 1:**
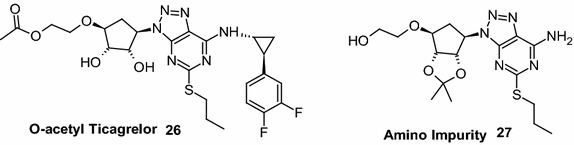
Potential impurities of ticagrelor

**Scheme 2:**
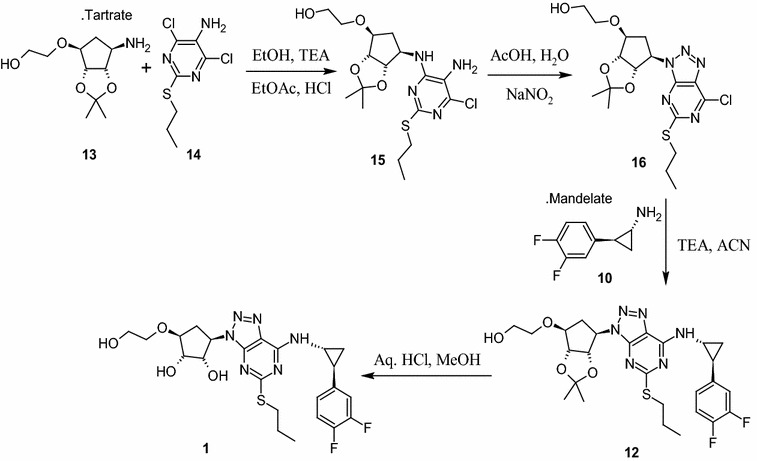
Improved process reported by innovator

The first synthetic approach reported (Hardern et al. 2003; Tadimeti and Chengzi 2011) for **1** is a multistep process (Scheme 1) and involve the reagents such as trifluoromethane sulfonyloxy acetic acid methyl ester in *n*-BuLi, isoamyl nitrite in bromoform, DIBAL-H in THF, and trifluoroacetic acid which are hazardous, toxic, flammable, pyrophoric and expensive. Further, the product formed by this method requires purification by column chromatography in order to control unacceptable amounts of critical and potential impurities formed in reaction and thus it is difficult to provide International conference on harmonization (ICH) grade material **1** suitable for formulation and thus process is not feasible at industrial scale. Diazotization of **15** with sodium nitrite in mixture of acetic acid and water yielded substituted triazole compound **16** with the formation of critical impurity **27** (Fig. 1) along with other impurities which are difficult to remove from product **1**. Further **16** was reacted with **10** in acetonitrile in presence of triethylamine to provide **12**, followed by deprotection of the diketal group of **12** with aqueous hydrochloric acid (aq. HCl) in methanol (MeOH) to provide **1** (Scheme 2) with 18.0 % yield and 98.25 % purity by HPLC.

Another synthetic approach reported (Ulf et al. 2001) by Larsson and his team made extensive efforts towards the convenient and efficient synthesis of **1**. Few of the steps adopted are superior, improved and scalable over the first reported syntheses. The improved process involve condensation of pyrimidine amine **14** with l-tartaric acid salt of **13** in ethanol in the presence of triethylamine at elevated temperature and high pressure (72 psi in autoclave) to provide **15**.

The reaction conditions involved in this approach for **15** requires pressure reactor, high temperature and longer reaction time (40–45 h) which is unsafe to handle at commercial scale. Moreover the product formed by this process requires column chromatographic purification to control critical impurities formed in the reaction, thus makes the process unsuitable for large scale production.

We hereby report a scalable, efficient, safe, and impurity-free process for the synthesis of ticagrelor (**1**) which is suitable for commercial production.

## Results and discussion

Process research and development of ticagrelor (**1**) presented in this article mainly focused on following four aspects: (a) to develop an efficient and high yielding process for the manufacture of one of the key intermediates **14** by establishing the safe critical process parameters for the nitration of **21** to achieve nitro compound **22**, (b) to avoid high pressure reaction for condensation of **13** and **14**, (c) to develop an alternative and safe diazotization process for preparing the triazole compound **16** using “Resin-NO_2_” which is safe to handle, reduces the formation of impurities and provide high reaction yield, and (d) to develop pure ticagrelor **1**, which is free from potential impurities **26** and **27** without the use of column chromatography purifications. The details are presented here.

### Development of an improved and safe process for **14**

**Scheme 3:**
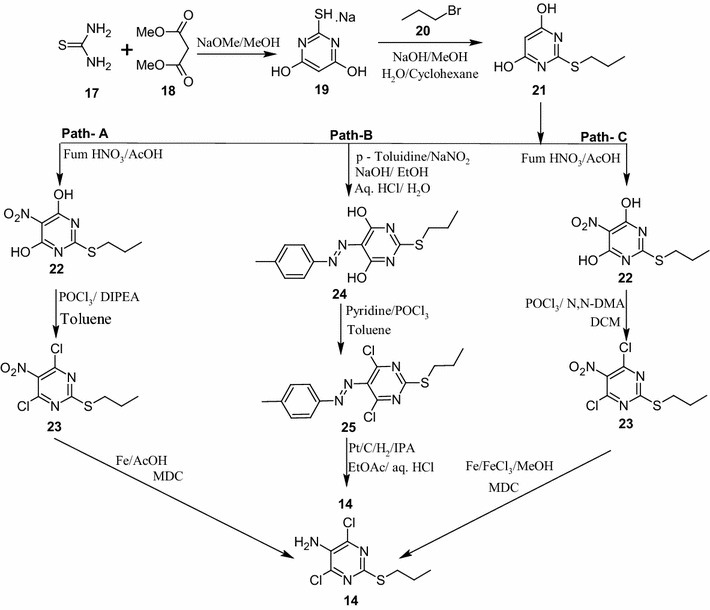
Synthetic approaches for the preparation of compound **14**

The thienopyrimidine **14** is one of the important building blocks required for the synthesis of ticagrelor (**1**). The literature processes (Scheme 3) for making **14** involves five steps wherein nitration and its reduction steps found to be critical and hazardous (Ulf et al. 2001; Khile et al. 2011). Reaction conditions used for nitration of **21** are found to be highly unsafe as we have observed several runaway kind of reaction during the initial experimentation in the laboratory. Further the reduction of obtained nitro compound **23** with Iron in the presence of acetic acid to yield **14** (Scheme 3, path A) posed serious operational problems as many of the reactions were again run away kind of nature with minor severity and lot of frothing. Filtration of unreacted iron and iron oxide with acetic acid was highly cumbersome as it took almost 2 days. The filtration of acetic acid in plant created irritant atmosphere and thus was not suitable. The reduction of diazo compound **25** to obtain amine **14** by hydrogenation in presence of Platinum catalyst on carbon according to path B (Scheme 3) though safe but the process was highly expensive and could not meet the economics.

For establishment of nitration of **21** we explored fuming nitric acid (alone) and fuming nitric acid along with acetic acid or sulfuric acid during feasibility experiments with precautionary temperature control during the addition or at the time of reaction progress but none of them worked out to be safe. We have then conducted DSC analysis for the nitration reaction to evaluate the safe reaction conditions. DSC analysis for the reaction mass was performed wherein mixture of fuming nitric acid and acetic acid was used as a nitrating agent. DSC thermogram shows exotherm at around 83 °C with heat evolution of 272 J/g of sample which corresponds to the exothermic temperature rise of around 219 °C, i.e. if reaction mass is heated up to 83 °C then temperature of the reaction mass could go up to 302 °C (83 + 219 °C) [ΔT]. Since ΔT was more than 50 °C the reaction is considered to be highly exothermic and thus the reaction needs to be conducted below 33 °C (83 − 50 = 33 °C) to avoid safety issues due to high exotherm. During laboratory optimization it has also been noticed that the dilution of the reaction mass with acetic acid minimized the exotherm to some extent thus we decided to perform nitration using fuming nitric acid along with acetic acid (5.0 times with respect to wt. of **21**) as the nitrating mixture at 0–5 °C. With these two critical reaction parameters we could perform the reaction safely during the commercial scale synthesis of **22** at 50 kg level. More importantly cooling/chilling unit of the plant should be efficient and it should not be allowed to break down during the reaction.

Further, the reduction of **23** has been developed with simple, inexpensive and mild reducing agent such as iron and catalytic quantity of ferric chloride [Fe/FeCl_3_] in presence of methanol at 50–55 °C (Gabriele et al. 2001). Reaction was smooth and went to completion within 3–4 h by HPLC. After completion of reaction, separation of the catalyst by filtration followed by acid–base workup of the filtrate provided amino compound **14** in 76 % yields and 99 % purity by HPLC (path C). Drastic change in the behavior of reaction and its filtration pattern was observed when Fe-FeCl_3_ was used as a reducing agent.

### Preparation of ticagrelor **1**

#### Condensation of **13** with **14** to provide compound **15**

During development, nucleophilic aromatic substitution of **13** with **14** has been attempted in various solvents such as ethanol, toluene, acetonitrile, and monoethylene glycol along with different organic and inorganic bases. The experiments conducted with inorganic bases such as Na_2_CO_3_, NaHCO_3_, K_2_CO_3_ Cs_2_CO_3_ and Li_2_CO_3_ with or without catalysts at different temperatures did not proceed to form compound **15** in the reaction whereas use of selected organic bases such as triethylamine (TEA), *N*,*N*-dimethyl aniline (DMA), diisopropyl ethyl amine (DIPEA) provided encouraging results as presented in Table 1.

**Table 1:** Screening experimental data for condensation of **13** and **14**

Solvent	Base	Catalyst	Temp (°C)	Time (h)	Reaction yield (%)	Isolated yield (%)	HPLC purity
Ethanol	TEA	–	75–78	30.0	62.5	28.25	68.00
IPA	DIPEA	DBU	78–80	50.0	70.2	34.28	75.00
Methanol	DIPEA	DBU	61–65	52.0	12.0	45.71	15.00
Toluene	TEA	KI	108–111	31.0	11.5	48.97	15.00
MEG	DIPEA	KI	120–125	6.0	83.5	72.00	99.51
MEG	DIPEA	–	120–125	5.0	85.00	67.97	99.60
MEG	DIPEA	DBU	120–125	5.0	85.5	77.14	99.65
MEG	TEA	DBU	120–125	6.0	54.0	54.29	60.00
ACN	K_2_CO_3_	–	80–85	30.0	25.0	60.00	30.00
DMF	K_2_CO_3_	–	100–105	24.0	16.0	56.00	21.00

Data clearly indicates that the reaction performed with MEG as solvent, DIPEA as a base and 1,8-diazabicyclo[5.4.0]undec-7-ene (DBU) as a catalyst provided very good reaction yield without applying the pressure (Table 1, entry 7). Further optimization to establish the mole ratios of **13** (with respect to **14**) using monoethylene glycol (MEG) as solvent, DIPEA as base and DBU as a catalysts provided encouraging results at 120–125 °C (Table 2).

**Table 2:** Optimization of mole ratio of compound **13** with respect to compound **14**

Compd. **14** (g)	Compd. **13** (mol. eq)	Base	Catalyst	Temp (°C)	Reaction yield (%)	Isolated yield (%)	HPLC purity (%)
10.0	1.00	DIPEA	DBU	120–125	85.15	59.40	99.03
10.0	0.90	DIPEA	DBU	120–125	82.30	54.25	95.11
10.0	1.05	DIPEA	DBU	120–125	87.95	69.85	99.38
10.0	1.10	DIPEA	DBU	100–105	88.65	67.14	98.38
10.0	1.20	DIPEA	DBU	120–125	94.50	73.14	99.31
25.0	1.10	DIPEA	KI	120–125	91.85	73.00	99.51
10.0	1.10	DIPEA	–	120–125	89.45	67.97	99.60
10.0	1.10	DIPEA	DBU	135–140	92.20	72.00	99.30
100.0	1.10	DIPEA	DBU	120–125	94.90	79.95	99.37
100.0	1.10	DIPEA	DBU	120–125	95.05	80.81	99.35
100.0	1.10	DIPEA	DBU	120–125	94.65	79.96	99.57

All the reactions are performed in MEG. 10 volumes of MEG was used w. r. to compound **13** provided favourable results

**Scheme 4:**
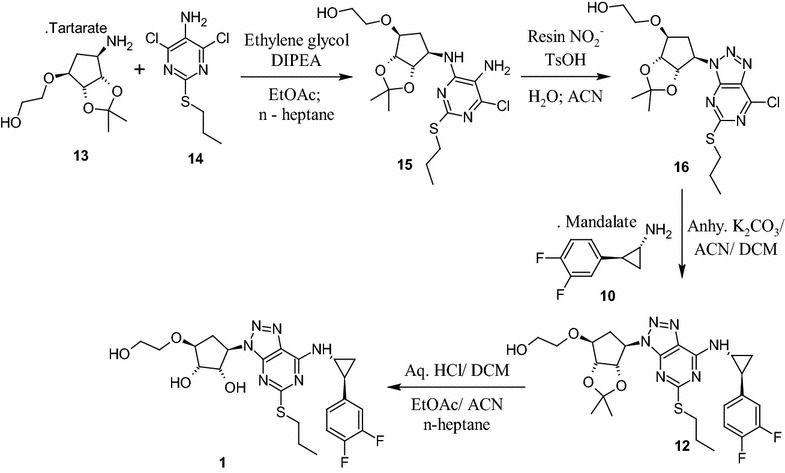
Improved approach for the synthesis of Ticagrelor (**1**)

After complete optimization, three consecutive experiments were performed with optimized parameters to check the consistency of the results with 100 g input of compound **14** (Table 2, entry nos. 9–11). After completion of reaction, the reaction mass was diluted with water, extracted the contents into ethyl acetate, ethyl acetate layer was washed with water, brine and concentrated to obtain the residue. Crystallization of the residue in ethyl acetate and *n*-heptane provided crystalline compound **15** with around 80 % yield and 99.5 % purity by HPLC. Thus a suitable process avoiding the pressure reactor, higher temperature, longer reaction times (40–45 h) and purification of the product by column chromatography has been developed (Scheme 4). The structure of crystalline compound **15** was characterized by analytical and spectroscopic methods. As compound **15** was isolated as a solid for the first time in our hands, the PXRD analysis was performed on the dried solid to understand the crystal pattern or polymorphic form (Mathad et al. 2014).

Further, the construction triazole ring on **15** to provide **16** comprises diazotization reaction followed by cyclization. The reported reactions for diazotization (NaNO_2_-acetic acid) involve highly acidic conditions that are found to be not suitable as it leads to deprotection compound **15** and formation of other unknown impurities which are difficult to remove. The alternative diazotizing agents such as alkyl nitrite (isoamyl nitrite in bromoform) is not suitable for diazotization on an industrial scale, since they are expensive, decompose during storage, corrosive and requires exclusively organic medium (ether, acetonitrile, THF, etc.,). Diazonium salts obtained under classical conditions are potentially explosive substances and thus need a very safer mechanism to handle in the plant scale. Diazotization using cation exchange resin along with mild acidic reagent such as *P*-toluene sulphonicacid (TsOH) with sodium nitrite in acetonitrile and water mixture releases brown fumes of gaseous nitrogen oxide which is again not safe in the production environment (Filimonov et al. 2008; Gorlushko et al. 2008; Lee et al. 2010; Moon et al. 2010). While exploring safer means of conducting this reaction, we have identified and introduced the anion exchange resin (Resin NO_2_^−^) saturated with nitrite ions from sodium nitrite successfully as diazotization reagent (Trusova et al. 2011) for synthesis of stable **16**. Resin-NO_2_^**−**^ was found to be an industrially feasible, safe and green reagent for the diazotization of **15**. The resin NO_2_^−^ was prepared by treating readily available Amberlyst A26 hydroxide form (Aldrich) with sodium nitrite solution in water. The suspension was filtered, washed with water till the filtrate solution shows neutral pH. The ion exchange capacity of the reagent was measured by potentiometer and found to be around 2.6–3.6 mmol of NO_2_^−^/g. The reagent was also found to be stable for at least 1 month in moist state. The diazotization process comprises a very simple and friendly operation; the compound **15** was stirred with Resin-NO_2_^−^ in water and acetonitrile (1:1) mixture in presence of TsOH for 20–30 min. After completion of the reaction, resin was separated by filtration followed by water washing. Compound **16** was extracted from filtrate layer into DCM and then DCM layer was washed with NaHCO3 solution and concentrated to yield compound **16** as thick syrup which was directly used for next reaction without further purifications. The resin obtained was recovered by treating it with sodium hydroxide (NaOH) solution to yield hydroxyl resin and it can be recycled further for diazotization by converting it to nitrite resin using the above process. Thus, a green and safe approach for diazotization of **15**, which produces no toxic gases and can be performed in water acetonitrile mixture at room temperature, is developed.

The next step of the process includes nucleophilic substitution of **16** with **10** to provide protected ticagrelor **12**. During development, reaction of **16** with **10** has been explored with various solvents in presence of selected bases at different temperature range. Among those combinations, reaction conducted in acetonitrile in presence of anhydrous K_2_CO_3_ at 25–30 °C for 2–3 h provided **12** as an oil with 98 % yield and 93 % purity by HPLC. The reaction with organic bases such as TEA and DIPEA though found good but taken longer reaction time (12–15 h) for the completion. The deprotection of ketal group on **12** was explored in various solvent like methanol, ethanol and DCM in presence of acidic reagents like acetic acid, trifluoro acetic acid and aq. HCl. Trifluroacetic acid reported for this reaction by earlier investigators was avoided as it was unsafe to handle in the production environment. Further, we have avoided the combination of methanol and aqueous HCl, wherein formation of potential genotoxic impurity, methyl chloride (CH_3_Cl) was observed at trace level in the product. It was also observed that the reaction mass exposed to strong acidic condition for longer time, generated degradation impurity (1*S*,2*S*,3*R*,5*S*)-3-[7-amino-5-(propylthio)-3*H*-[1,2,3]triazole [4,5-*d*]pyramid -in-3-yl]-5-(2-hydroxyethoxy)-1,2-cyclopentanediol impurity (**27**) both in methanol and ethanol. When reaction was performed in DCM and aq. HCl (biphasic condition), reaction was proceeded smoothly in 2–3 h with reduced amount of impurities (less than 0.05 % by HPLC) due to less contact time of the product with acid. Hence, DCM has been selected as a reaction medium and aq. HCl as an acid source.

After completion of the reaction, the reaction mass was cooled to 10 °C and adjusted the pH of the contents to 9–11 with NaOH solution followed by distillation of DCM below 40 °C to provide the oily syrup. The syrup was then diluted with water, extracted from ethyl acetate, and ethyl acetate layer was concentrated to provide product **1** as syrup. The syrup was then recrystallized in neat acetonitrile to furnish **1** as a white crystalline solid with an overall yield of 63 % and HPLC purity of 99.85 % (Scheme 4; Table 3). The potential O-acetyl ticagrelor impurity **26** formed during the work-up was found below 0.10 % and degraded impurity **27** below 0.05 % by HPLC. The impurity profile data of three consecutive batches was provided in Table 3.

**Table 3:** Monitoring the elimination of impurities in downstream process for **1**

Sample station (for HPLC analysis)	Impurities and there content by HPLC (%)					
	**1**	**12**	**16**	**26**	**27**	SMUI
Crude	99.25	ND	ND	0.32	0.01	0.06
Acetonitrile purification	99.74	ND	ND	0.05	0.01	0.06
Crude	98.70	0.07	ND	0.30	0.06	0.14
Acetonitrile purification	99.60	ND	ND	0.05	0.01	0.05
Crude	99.44	ND	ND	0.29	0.02	0.03
Acetonitrile purification	99.78	ND	ND	0.04	ND	0.03

*ND* not detected

## Conclusion

In conclusion, simple, efficient, production friendly and environment friendly green chemistry approach process for the synthesis of ticagrelor that is substantially free from potential impurities **26** and **27**.

## Experimental section

Melting points were determined on Analab melting point apparatus, in open capillary tubes and are uncorrected. The ^1^H NMR (400 MHz) and ^13^C NMR (100 MHz) spectra were recorded on a Varian Gemini 400 MHz FT NMR spectrometer. Chemical shifts were reported in parts per million (ppm) using tetramethylsilane as internal standard and are given in δ units. The solvents used for NMR spectra were deuterated chloroform and deuterated dimethylsulfoxide unless otherwise stated. Infrared spectra were taken on Perkin Elmer Spectrum 100 in potassium bromide pallets unless otherwise stated. Elemental analyses were performed on a Hosli CH-Analyzer and the results were within ±0.3 % of the calculated values. High-resolution mass spectra were obtained with a Shimadzu GC–MS QP mass spectrometer with an ionization potential of 70 eV. All the reaction were monitored by thin layer chromatography (TLC), carried out on 0.2 mm silica gel 60F254 (Merck) plates using UV light (254 and 366 nm) or High performance liquid chromatography (HPLC) on Agilent Technologies 1200 series for detection. Gas chromatography on Agilent Technologies 7683B with head space was used for analyzing the residual solvents. Common reagent–grade chemicals are either commercially available and were used without further purification or were prepared by standard literature procedures.

### 2-[{(3a*R*,4*S*,6*R*,6a*S*)-6-{[5-amino-6-chloro-2-(propylthio)-4-pyrimidinyl] amino}-2,2-dimethyltetrahydro-3a*H*-cyclopenta[*d*][1,3]dioxol-4-yl)}oxy]-1-ethanol, (**15**)

To a stirred solution of **14** (1.0 kg, 4.19 mol) in ethylene glycol 5.0 L with **13** (1.69 kg, 4.61 mol) was added DBU (50.0 g), followed by addition of DIPEA (2.43 kg, 18.85 mol) at 25–30 °C. The reaction temperature was slowly raised to 120–125 °C and maintained for 4–5 h. After completion of reaction (by HPLC), reaction mass was cooled to 25–30 °C and diluted with water (20 L). The pH of reaction mass was adjusted to 4–5 with aqueous hydrochloric acid and product was extracted with ethyl acetate (15 L). The ethyl acetate layer was separated, washed with water (10 L) and 15 % (w/v) sodium chloride solution (10 L). Collected ethyl acetate layer was concentrated under reduced pressure at 50–55 °C to provide thick syrup (1.6 kg). The syrup was dissolved in ethyl acetate (1 L) stirred, heated at 50 °C, followed by addition of *n*-heptane (20 L) at 50 °C and maintained for 30 min. The resulting isolated suspensions was gradually cooled, chilled at 0–5 °C, maintained for 30–40 min; obtained solid was filtered, washed with *n*-heptane (1 L), suck dried and dried at 50–55 °C to afford **15** as white crystalline solid. Yield: 1.41 kg, (80.53 %). Purity by HPLC: 99.51 %. M.P: 108–112 °C. IR (KBr, cm^−1^): 3441.88, 3379.74, 3246.07, 2935.63, 1644.23, 1570.57, 1373.35, 1115.91, 1050.31, 951.55, and 763. 48. PXRD (2θ°): 5.36, 6.65, 10.53, 13.39, 18.21, 22.54, 24.18. The spectral data of compound **15** prepared by our process were found to be in agreement with the reported data (Tadimeti and Chengzi 2011).

*Resin NO*_2_^−^ To a stirred solution of sodium nitrite (0.6 kg) in water was added Amberlyst A26 hydroxide form (1.0 kg). The reaction mass was stirred for 25–30 min at 25–30 °C. Resin was filtered and washed with water (3 × 2 L) till the pH of filtrate shows 7–8 and suck dried the material for 10–15 min to provide resin NO_2_^−^ as brown granules. Yield: 0.95 kg, (95 %); Ion exchange capacity: 2.6–3.6 mmol of NO_2_^−^/g.

### 2-[{(3a*R*,4*S*,6*R*,6a*S*)-6-[7-chloro-5-(propylthio)-3*H*-[1,2,3]triazolo[4,5-*d*] pyrimidin-3-yl]-2,2-dimethyltetrahydro-3a*H*-cyclopenta[*d*][1,3]dioxal-4-yl}oxy]-1-ethanol (**16**)

To a stirred solution of TsOH (0.68 kg, 3.58 mol) in water (5 L) was added resin nitrite (2.0 kg) and reaction mass was stirred for 10–15 min followed by addition of **15** (1.0 kg, 2.38 mol) in acetonitrile (5 L). The reaction mass was stirred for 20–30 min at 25–30 °C. Upon completion of reaction by TLC, resin was removed by filtration and washed with DCM (10 L). The mother liquor was combined and diluted with water (10 L). The DCM layer was separated, washed with water (10 L) followed by 15 % (w/v) sodium chloride solution and evaporated under reduced pressure at 35–40 °C to provide **16** as thick syrup. Yield: 1.01 kg, (98.42 %). HPLC purity: 92.0 %. The spectral data of compound **16** prepared by our process were found to be in agreement with the reported data (Tadimeti and Chengzi 2011).

### 2-[{(3a*R*,4*S*,6*R*,6a*S*)-6-[7-{[(1*R*,2*S*)-2-(3,4-diflurophenyl)-cyclopropyl]amino}-5(propylthio)-3*H*-[1,2,3]triazolo[4,5-*d*]pyrimidin-3-yl]-2,2-dimethyltetrahydro-3a*H*-cyclopenta[*d*][1,3] dioxal-4-yl}oxy]-1-ethanol (**12**)

To a stirred solution of **16** (1.0 kg, 2.32 mol) in acetonitrile (7 L) was added **10** (0.709 kg, 2.2 mol) and anhydrous K_2_CO_3_ (0.640 kg, 4.64 mol) and stirred at 25–30 °C for 2–3 h. After completion of the reaction by HPLC, reaction mass was diluted with water (10 L) and extracted with DCM (10 + 5 L). DCM layer was separated, washed with water (10 L) followed by 15 % (w/v) sodium chloride solution (10 L) and concentrated under reduced pressure at 35–40 °C to provide **12** as thick brownish syrup. Yield: 1.25 kg, (95.52 %). HPLC purity: 93.0 %. The spectral data of compound **12** prepared by our process were found to be in agreement with the reported data (Tadimeti and Chengzi 2011).

### (1*S*,2*S*,3*R*,5*S*)-3-[7-{[(1*R*,2*S*)-2-(3,4-Difluorophenyl)cyclopropyl]amino}-5-(propylthio)-3*H*-[1,2,3]-triazolo[4,5-*d*]pyrimidin-3-yl]-5-(2-hydroxyethoxy)-1,2-cyclopentanediol (ticagrelor, **1**)

To a stirred solution of **12** (1.0 kg, 1.77 mol) in DCM (7 L), was added aqueous hydrochloric acid (5 L) and the resultant reaction mass was stirred for 2–3 h. Upon completion of reaction by HPLC, the pH of reaction mass was adjusted to 9–11 with aqueous NaOH solution (~2.4 L). The DCM layer was concentrated under reduced pressure to provide oil. The oil was diluted with water (12 L) and extracted in ethyl acetate (2 × 10 L). Combined ethyl acetate layer was washed with 2 % (v/v) hydrochloric acid solution (10 L), 15 % (w/v) NaHCO_3_ solution and further by 10 % (w/v) sodium chloride solution (2 × 10 L). The ethyl acetate layer was then concentrated under reduced pressure at 50–55 °C to provide thick syrup. The obtained syrup was again dissolved in acetonitrile (4.5 L) at 55–60 °C and then cooled to 5–10 °C and stirred for 1–2 h. The solid product obtained was filtered and dried to provide **1** as white crystalline solid. Yield: 0.76 kg, (81.18 %). HPLC purity: 99.85 %. The spectral data of compound **1** prepared by our process were found to agree with the reported data (Tadimeti and Chengzi 2011).

### (1*S*,2*S*,3*R*,5*S*)-3-[7-{[(1*R*,2*S*)-2-(3,4-Difluorophenyl)cyclopropyl]amino}-5-(propylthio)-3*H*-[1,2,3]-triazolo[4,5-*d*]pyrimidin-3-yl]-5-(2-*O*-acetylhydroxyethoxy)-1,2-cyclopentanediol (**26**)

To a stirred solution of ticagrelor (**1**, 10.0 g, 0.018 mol) in ethyl acetate (200 cm^3^) was added concentrated hydrochloric acid (10 cm^3^). The reaction mass was heated at 55–60 °C and stirred for 5–6 days to get maximum conversion to product, reaction mass was cooled to room temperature followed by filtration to get crude product. Crude product obtained was purified by column chromatography using silica gel as stationary phase with toluene and acetone as eluent (9:1 ratio) to offer **26** as a white crystalline solid upon evaporation of solvent. Yield: 6.7 g, (62.03 %). HPLC purity: 97.5 %. M.P: 124.5–125 °C. IR (KBr, cm^−1^): 3261.02, 2965.41, 1741.62, 1621.59, 1522.02 and 1050.31. MS *m*/*z* (%): 565.0 [M^+^ + 1] (100). ^1^H NMR (DMSO-*d*_*6*_): *δ* 9.37–9.36 (d, 1H), 7.37–7.24 (q, 2H), 7.08–7.07 (d, 1H), 5.16–5.14 (d, 1H), 5.09–5.08 (d, 1H), 4.99–4.52 (t, 1H), 4.56–4.51 (q, 1H), 4.13–4.10 (t, 2H), 3.93–3.91 (t, 1H), 3.78–3.75 (t, 1H), 3.70–3.62 (m, 2H), 3.17–3.13 (q, 1H), 2.96–2.89 (q, 1H), 2.87–2.80 (q, 1H), 2.67–2.59 (q, 1H), 2.14–2.09 (m, 1H), 2.02–1.98 (t, 4H), 1.55–1.46 (m, 3H), 1.39–1.36 (t, 1H), 0.83–0.79 (t, 3H). ^13^C NMR (DMSO-*d*_*6*_): *δ* 170.45, 169.22, 168.35, 155.44, 154.00, 150.73, 149.43, 149.09, 148.30, 146.68, 139.37, 123.23, 122.87, 117.16, 114.95, 81.80, 74.34, 73.78, 66.86 (CH_2_), 63.37 (CH_2_), 60.70, 35.74, 34.14, 33.09 (CH_2_), 32.36 (CH_2_), 24.09, 24.09, 22.36 (CH_2_), 20.76, 16.59 (CH_2_), 15.05 (CH_2_), 13.01 (CH_3_).

### (1*S*,2*S*,3*R*,5*S*)-3-[7-amino-5-(propylthio)-3*H*-[1,2,3]triazolo[4,5-*d*]pyrimidin-3-yl]-5-(2 hydroxyethoxy)-1,2-cyclopentanediol (**27**)

To a stirred solution of **16** (10.0 g, 0.025 mol) in tetrahydrofuran (100 cm^3^) was added ammonium hydroxide (100 cm^3^) and stirred at 25–30 °C for 7–8 h. After completion the reaction by TLC, reaction mass was filtered, washed with tetrahydrofuran (10 cm^3^), and dried under vacuum to provide **27** as white crystalline solid. Yield: 7.95 g, (83.68 %). HPLC purity: 98.2 %. M.P: 175.8–176.9 °C. IR (KBr, cm^−1^): 3413.4, 3178.2, 2961.9, 1665.7, 1571.0, 1321.5, 1121.1. MS *m*/*z* (%): 371.2 [M^+^ + 1] (100). ^1^HNMR (DMSO-*d*_*6*_): *δ* 8.41 (bs, OH), 8.06 (bs, OH), 5.36 (bs, NH_2_), 4.99–4.92 (d, 1H), 4.64 (bs, OH), 4.59–4.55 (q, 1H), 3.94–3.93 (d, 1H), 3.76–3.73 (t, 1H), 3.51–3.45 (q, 4H), 3.13–3.01 (m, 2H), 2.65–2.57 (m, 1H), 2.08–2.01 (m, 1H), 1.72–1.65 (q, 2H), 1.59 (s, 1H), 1.00–0.96 (t, 3H). ^13^C NMR (DMSO-*d*_*6*_): *δ* 169.08, 155.14, 149.66, 122.86, 81.82, 74.19, 73.72, 70.82 (CH_2_), 60.86, 60.28 (CH_2_), 33.10 (CH_2_), 32.14 (CH_2_), 25.22, 22.44 (CH_2_), 13.30 (CH_3_).

### Sodium salt of thiobarbutaric acid (**19**)

Sodium methoxide (70.94 g; 1.31 mol) was added to stirred and hot solution of thiourea (**17**, 100.0 g, 1.31 mol) and dimethyl malonate (**18**, 208.26 g, 1.57 mol) in methanol (200 cm^3^) at 50–55 °C and maintained for 3–4 h. Upon completion of reaction, the reaction mass was cooled to 0–5 °C and maintained for 30–45 min. The product obtained was filtered, washed with pre-chilled methanol (25 cm^3^) and dried under vacuum at 50–55 °C for 2–3 h to offered sodium salt of **19** as an white crystalline solid. Yield: 190.0 g, (88.0 %). HPLC purity: 99.7 %. The spectral data of compound **19** prepared by our process were found to be in agreement with the reported data (Tadimeti and Chengzi 2011).

### 2-(propylthio)pyrimidine-4,6-diol (**21**)

To a stirred solution of NaOH (59.83 g, 1.50 mol) in water (300 cm^3^) was added **18** (100.0 g, 0.59 mol) and propyl bromide (**20**, 88.29 g, 0.72 mol). The reaction mass was heated at 50–55 °C and maintained for 4–5 h. After completion of reaction by TLC, the reaction mass was diluted with water (300 cm^3^) and acidified with aqueous hydrochloric acid till pH 0.5–1.5. The contents were stirred for 60–90 min and the solid obtained was filtered, washed with water (100 cm^3^) and dried under vacuum at 55–60 °C for 4–5 h to provide **21** as white crystalline solid. Yield: 105.0 g, (80.0 %). HPLC purity: 97.5 %. The spectral data of compound **21** prepared by our process were found to be in agreement with the reported data (Tadimeti and Chengzi 2011).

### 5-Nitro-2-(propylthio)pyrimidine-4,6-diol (**22**)

To a pre chilled (0–5 °C) solution of acetic acid (500 cm^3^) was added fuming nitric acid (83.89 g, 1.33 mol) and stirred for 10–15 min. Compound **21** (100.0 g; 0.54 mol) was added lot wise (five equal lots) below 5 °C to the above nitrating mixture and maintained at 0–5 °C for 45–60. After completion of reaction by TLC, the reaction mass was diluted with water (500 cm^3^) and stirred for 45–60 min. The obtained solid was filtered, washed with water (300 cm^3^) and dried under vacuum at 55–60 °C for 4–5 h to afford **22** as crystalline pale yellow solid. Yield: 95.0 g, (80.0 %). HPLC purity: 98.4 %. The spectral data of compound **22** prepared by our process were found to be in agreement with the reported data (Tadimeti and Chengzi 2011).

### 4,6-Dichloro-5-nitro-2-(propylthio)pyrimidine (**23**)

To a pre cooled (0–5 °C) mixture of **22** (100.0 g, 0.43 mol) and phosphorus oxy chloride (238.71 g, 1.55 mol) was added *N*,*N*-dimethyl aniline (104.8 g, 0.86 mol). The mixture was heated at 100–110 °C and maintained for 60–90 min. After completion of reaction by TLC the reaction mass was cooled at 25–30 °C and then quenched over chilled water (1.5 L). The product was extracted in DCM (2 × 500 cm^3^) and the DCM layer was washed with 10 % (w/v) sodium bicarbonate solution (500 cm^3^) followed by 15 % (w/v) sodium chloride solution (500 cm^3^). Concentration of DCM under reduced pressure at 35–40 °C furnished **23** as brownish oil. Yield: 111.0 g, (95.73 %). HPLC purity: 97.80 %. The spectral data of compound **23** prepared by our process were found to be in agreement with the reported data (Tadimeti and Chengzi 2011).

### 4,6-Dichloro-5-amino-2-(propylthio)pyrimidine (**14**)

To a stirred solution of **23** (100.0 g, 0.37 mol) in methanol (800 cm^3^) was added iron powder (116.62 g, 2.08 mol) and the contents were heated up to 35–40 °C. Ferric chloride (13.0 g, 0.08 mol) solution in water (130 cm^3^) was added to the above mixture and heated at 50–55 °C for 3–4 h. Upon completion of reaction by TLC, the reaction mass was cooled to 25–30 °C and iron was removed by filtration. The collected filtrate was concentrated under reduced pressure to yield oil which was diluted with water (500 cm^3^), basified with ammonium hydroxide and extracted with DCM (500 cm^3^). DCM layer was washed with 10 % (w/v) EDTA solution (500 cm^3^) followed by 15 % (w/v) sodium chloride solution (500 cm^3^) and evaporated at 35–40 °C under reduced pressure to provide **14** as crude oil. The crude oil was diluted with di-isopropyl ether (500 cm^3^) and pH of the solution was adjusted to 0.5–1.5 using concentrated hydrochloric acid (50 cm^3^). Diisopropyl ether layer was separated, washed with water (2 × 500 cm^3^) and evaporated under reduced pressure at 45–50 °C to offer **14** as brownish thick oil. Yield: 72.0 g, (81.07 %). HPLC purity: 99.60 %. IR (NaCl, cm^−1^): 3473.83, 2964.84, 1608.13, 1500, 1402.55, 1093.74. MS *m*/*z* (%): 238.1 [M^+^ + 1] (100). ^1^HNMR (CDCl_3_): *δ* 4.19 (bs, NH_2_), 3.08–3.05 (t, 2H) 1.77–1.67 (m, 2H), 1.03–1.00 (t, 3H). ^13^C NMR (CDCl_3_): *δ* 158.25, 145.06, 131.39, 33.20 (CH_2_), 22.24 (CH_2_), 13.29 (CH_3_).

## References

[CR1] Filimonov VD, Semenischeva NI, Krasnokutskaya EA, Tretyakov AN, Hwang HY, Chi KW (2008). Sulfonic acid based cation-exchange resin: a novel proton source for one-pot diazotization–iodination of aromatic amines in water. Synthesis.

[CR2] Gabriele B, Salerno G, Veltri L, Costa M, Massera C (2001). Stereoselective synthesis of (E)-3-(methoxycarbonyl)methylene-1,3-dihydroindol-2-ones by palladium-catalyzed oxidative carbonylation of 2-ethynylanilines. Eur J Org Chem.

[CR3] Gorlushko DA, Filimonov VD, Semenishcheva NI, Krasnokutskaya EA, Tretyakov AN, Chi KW (2008). A simple and efficient procedure for diazotization–iodination of aromatic amines in aqueous pastes by the action of sodium nitrite and sodium hydrogen sulfate. Russ J Org Chem.

[CR4] Haberfeld H (ed) (2010) Austria-Codex (in German) (2010/2011 edn). Österreichischer Apothekerverlag, Vienna

[CR5] Hardern D, Ingall A, Springthorpe B, Willis P, Guile S (2003) Triazolo(4,5-d)pyrimidine compounds. US Patent 6,525,060 B1, Feb 15, 2003

[CR6] Huber K, Hamad B, Kirkpatrick P (2011). Ticagrelor, an oral antiplate therapy. Nat Rev Drug Discov.

[CR7] Jacobson KA, Boeynaems JM (2010). P2Y12 Nucleotide receptors: promise of therapeutic applications. Drug Discov Today.

[CR8] Khile AS, Patel J, Trivedi N, Pradhan NS (2011) Improved process for preparing ticagrelor intermediate, 4,6-dichloro-5-nitro-2-(propylthio) pyrimidine. PCT Int Appl WO 11/101740, Aug 25, 2011

[CR9] Lee YM, Moon ME, Vajpayee V, Filimonov VD, Chi KW (2010). Efficient and economic halogenation of aryl amines via arenediazonium tosylate salts. Tetrahedron.

[CR10] Mathad VT, Niphade NC, Shinde GB, Padaki SA, Mahale PK (2014) A process for preparation of ticagrelor and intermediate thereof. PCT Int Appl WO 14/102830 A1, July 3, 2014

[CR11] Moon ME, Choi Y, Lee YM, Vajpayee V, Trusova M, Filimonov VD, Chi KW (2010). An expeditious and environmentally benign preparation of aryl halides from aryl amines by solvent-free grinding. Tetrahedron Lett.

[CR12] Springthorpe B, Bailey A, Barton P, Birkinshaw TN, Bonnert RV, Brown RC, Chapman D, Dixon J, Guile SD, Humphries RG, Hunt SF, Ince F, Ingall AH, Kirk IP, Leeson PD, Leff P, Lewis RJ, Martin BP, McGinnity DF, Mortimore MP, Paine SW, Pairaudeau G, Patel A, Rigby AJ, Riley RJ, Teobald BJ, Tomlinson W, Webborn PJH, Willis PA (2007) From ATP to AZD6140: the discovery of an orally active reversible P2Y12 receptor antagonist for the prevention of thrombosis. Bioorg Med Chem Lett 17:6013 10.1016/j.bmcl.2007.07.057 17827008

[CR13] Tadimeti R, Chengzi Z (2011) Cyclopropyl modulators of P2Y12 receptors. PCT Int Appl WO 11/017108 A2

[CR14] Trusova ME, Krasnokutskaya EA, Postnikov PS, Choi Y, Chi W, Filimonov V (2011). A green procedure for the diazotization–iodination of aromatic amines under aqueous, strong acid free condition. Synthesis.

[CR15] Ulf L, Mattias M, Tibor M, Andreas P (2001) Novel triazolo pyrimidine compounds. PCT Int Appl WO 01/92263 A1, Dec 6, 2001

[CR16] Van Giezen JJ, Humphries RG (2005). Preclinical and clinical studies with selective reversible direct P2Y12 antagonists. Semin Thromb Hemost.

[CR17] Zhang H, Liu J, Zhang L, Kong L, Yao H, Sun H (2012). Synthesis and biological evaluation of ticagrelor derivatives as novel antiplatelet agents. Bioorg Med Chem Lett.

